# Papillary Carcinoma in Mature Teratoma of Struma Ovarii

**DOI:** 10.5334/jbr-btr.855

**Published:** 2015-09-15

**Authors:** D. Srbovan, J. Mihailović, K. Nikoletić, E. Matovina, N. Šolajić

**Affiliations:** 1 Department of Nuclear Medicine, Institute of Oncology Vojvodina, Sremska Kamenica, Serbia; 2 Clinic of Operative Oncology – Department of Patohystology and Cytology Diagnostic, Institute of Oncology Vojvodina, Sremska Kamenica, Serbia

**Keywords:** Teratoma

## Abstract

A 62-year-old woman had the incidental finding of malignant struma ovarii following surgery for primary endometrial carcinoma. The patient had vaginal bleeding for one year. After gynecological examination, she was referred for fractional curettage which revealed endometrial cancer. The patient underwent total hysterectomy and bilateral adnexectomy. Histological findings of uterus confirm the presence of endometrial cancer. The left ovary showed the presence of mature teratoma with dominant thyroid tissue and focus of papillary carcinoma. Postoperatively she underwent radiation therapy and 3 months later total thyroidectomy. The stimulated thyroglobulin level was detectable. She was referred for radioiodine ablation with a dose of 3,7GBq 131-J. Post therapy scintigraphy shows pathological uptake of 131-J only in the neck. The patient continued treatment of endometrial cancer (external beam therapy). She is currently on suppressive hormone L-thyroxin therapy. Two months later hormonal status, thyroglobulin and antithyroglobulin antibodies showed optimal range.

Struma ovarii is a rare form of germ cell derived ovarian tumor defined by the presence of thyroid tissue comprising more than 50% of the overall mass [1]. Struma ovarii was first described in 1899 which literally means “goiter of the ovary”. It represents 1% of all ovarian tumors [2]. The vast majority of struma ovarii are benign (95%); however, malignant tumors have been reported in a small percentage of cases [3].

Most commonly, they occur as part of a teratoma but may occasionally be encountered with serous or mucinous cystadenomas [4]. Germ cell tumors are derived from the primordial germ cells of the ovary. Twenty to twenty five percent of all benign and malignant ovarian neoplasms is of germ cell origin [5]. Most of these tumors are mature teratomas. Teratomas are composed of various tissue including hair, skin, bone, teeth, as well as thyroid. Struma ovarii is a rare monodermal variant of ovarian teratoma accounting for only 2% of all mature teratomas. It is a benign condition but in about 5% of cases, malignant transformation is observed [6,7,8]. Due to its rarity, there has been controversy about the diagnosis and treatment.

## Case report

**Figure 1:**
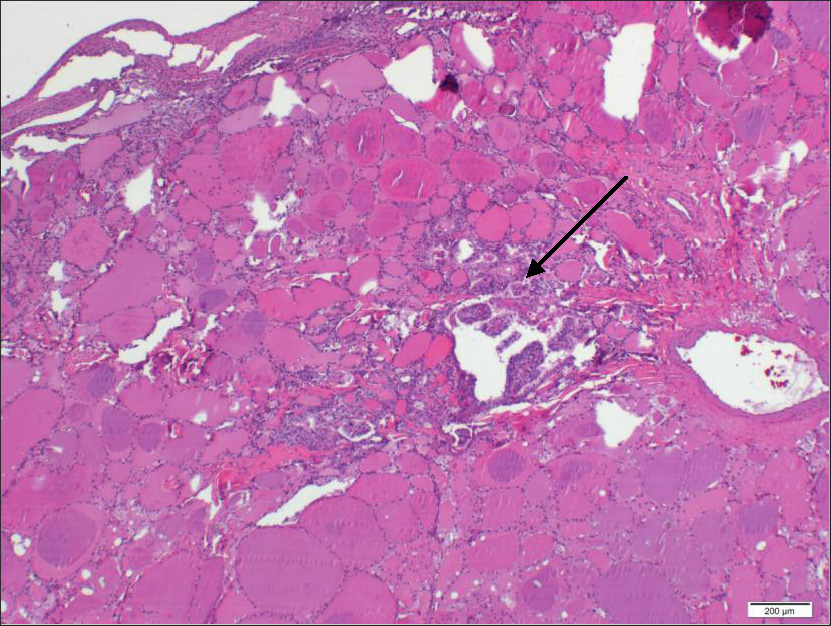
Microscopic focus of unencapsulated papillary carcinoma (1.3 mm in diameter) inside the thyroid tissue. H&E, 40x.

**Figure 2:**
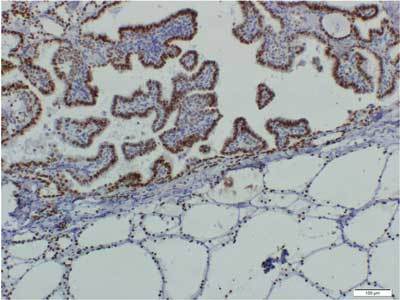
Diffuse nuclear immunoreactivity for TTF-1 in both benign thyreocytes and in the malignant ones. Immunoperoxidase with hematoxylin counterstain, 100x.

A 62-year-old woman was admitted to the Department of Gynecology at the Institute of Oncology Vojvodina with the complaint of vaginal bleeding for one year. Her past medical history was uneventful. The biopsy results of fractional curettage identified endometrial cancer (HP: carcinoma endometriodes, G2). She underwent total hysterectomy with bilateral adnexectomy. Histological findings confirmed the presence of endometrial cancer (HP: adenocarcinoma endometriodes endometrial, HG2, pT1c, FIGO Ic and Lieomyoma uteri). The right ovary was without pathological lesions, but the left ovary had a mature teratoma with dominant thyroid tissue and lesion of papillary cancer, 1,3 mm in diametar – malignant struma ovarii (Figs. 1, 2).

**Figure 3:**
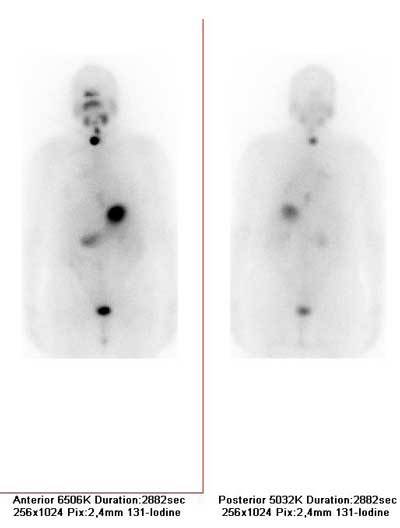
Postth whole body scan after aplication of radioiodine therapy in dose 3,7GBq 131-two foci of 131-J uptake in the neck.

Three months after completing brachytherapy, she underwent total thyroidectomy. Histological findings were without evidence of papillary cancer (HP: Struma colloides polynodosa glandule thyroideae).The stimulated thyroglobulin (tumor marker in histological confirmation of thyroid cancer) level was detectable (Tg, 8.8 ng/ml; TSH, 25.49 mIU/ml) and negative antithyroglobulin antibodies. We decided to apply the radioiodine therapy in a dose of 3,7GBq 131-J. Post therapy whole body scintigraphy did not show distant metastases. Two foci of 131-I uptake were seen in the neck (Fig. 3). The patient receives suppressive hormone L-thyroxin therapy. One month after radioiodine ablation, she continued treatment of endometrial cancer (external beam therapy). The first post therapy check of hormonal status, Tg and ATA were in an optimal range.

## Discussion

Struma ovarii is a very rare gynecologic tumor. Due to its rarity, there has been controversy about its diagnosis and treatment. There is no uniform criterion about diagnosis and treatment. Some authors suggest that following the pathological diagnosis of malignant struma ovarii, the same guidelines as those for thyroid carcinoma should be used [9].

The tumor can be characterized by radiological imaging; however, the final diagnosis is made based on pathological and histological examination of the tissue. Surgical resection remains the definitive treatment for benign disease. Surgery with adjuvant radioiodine therapy has shown to be successful in treating metastatic and recurrent disease [10]. Extra ovarian spread or later recurrence of struma ovarii is exceedingly rare [11].

This patient was treated surgically including total hysterectomy with bilateral adnexectomy as well as total thyroidectomy followed by radioiodine ablation therapy. With this therapy, primary thyroid carcinoma is excluded. Total 131-I body scan detects metastatic spread of disease. Serum thyroglobulin detects recurrence or lack of the disease.

## Conclusion

Since there are no guidelines for treatment of malignant struma ovarii, decisions should be made for each patient individually. There are multiple advantages to treating malignant struma ovarii with thyroid surgery and radioiodine therapy. This approach excludes primary thyroid carcinoma and enables use of 131-I and serum thyroglobulin for surveillance of possible recurrent disease.

## Competing Interests

The authors declare that they have no competing interests.
